# Cryptic diversity in the inshore hagfish, *Eptatretus burgeri* (Myxinidae, Pisces) from the northwest Pacific

**DOI:** 10.1080/23802359.2020.1823256

**Published:** 2020-09-22

**Authors:** Young Sun Song, Seung-Eun Bae, Jung-Ha Kang, Jung-Youn Park, Jin-Koo Kim

**Affiliations:** a Department of Marine Biology, Pukyong National University, Busan, Korea; b National Institute of Fisheries Science, Busan, Korea

**Keywords:** *Eptatretus burgeri*, cryptic diversity, evolutionary significant unit, management unit

## Abstract

The fishery of inshore hagfish (*Eptatretus burgeri*) is particularly important from the perspective of the eel-skin leather industry in the northwest Pacific. In order to reveal the genetic diversity and population structure of *E. burgeri* in the northwest Pacific, we analyzed partial nucleotide sequences of three mitochondrial DNA regions (523 bp in COI, 712 bp in ND4 and 617 bp in Cyt*b*) based on specimens collected from six locations in Korea and Japan. The genetic diversities of *E. burgeri* were higher in Korean locations compared to Japanese ones. AMOVA showed that *E. burgeri* was completely separated into two groups (group A: southern coast of Korea and western coast of Japan vs. group B: eastern coast of Japan). Furthermore, groups A and B were divided into each two lineages (lineage I: west southern coast of Korea, lineage II: east southern coast of Korea and western coast of Japan, lineage III and IV: eastern coast of Japan). Our molecular results suggest that these two groups and lineages of *E. burgeri* may be different evolutionary significant unit and management unit, respectively.

## Introduction

Hagfish has a high commercial value and significant economic importance from the perspective of the fisheries industry (eel-skin leather and food) in the northwest Pacific (Kase et al. 2018). Hagfish catches have greatly declined over the last decades in Korean and Japanese waters (McMillan and Wisner 2004; Knapp et al. 2011). *Eptatretus burgeri*, which is major commercial species, is found in the restricted patch range around the northwest Pacific such as Korea, Japan, and Taiwan (Mok and Chen 2001; Froese and Pauly 2019). Most in Korea is incidentally caught by fish traps for *Conger myriaster* and directly targeted by using longlines, and its production is approximately within 93 tons during a five-year period (MOF 2020). There are no regulations governing the harvesting of *E. burgeri*. Mitochondrial DNA (mtDNA) has proven to be useful for population genetic studies (Avise et al. 1987; Schultheis et al. 2002). To manage a sustainable *E. burgeri* fishery, we investigated the genetic diversity and population structure of Korean and Japanese *E. burgeri* using three Mitochondrial DNA (mtDNA) sequence markers.

## Materials and methods

### Sampling

A total of 180 specimens of *E. burgeri* were collected from Tongyeong (TY; *n* = 38; 34°39′1.21″N; 128°26′6.15″E), Wando Island (WD; *n* = 30; 33°59′56.5″N 126°53′57.0″E), Jeju Island (JJ; *n* = 33; 33°37′51.5″N 126°53′11.4″E) in Korean waters, and Kyoto (KT; *n* = 31; 35°40′29.1″N 135°27′03.4″E), Tsushima Island (TS; *n* = 30; 34°20′21.5″N 129°28′23.9″E), Shikoku Island (SK; *n* = 18; 32°47′19.2″N 132°32′55.6″E) in Japanese waters between May 2014 and Sep 2017, caught by hook and line, and fishing trap (Figure S1). The specimens have been deposited in the Marine Fish Resource Bank of Korea (MFRBK) at Pukyong National University (PKU), Korea. We loaned the Kyoto specimens and received the Shikoku Island specimens from Kyoto University (FAKU).

### DNA extraction, PCR and sequencing, and data analysis

Genomic DNA was extracted from the muscle tissues using DNA Extraction Kit (Bioneer Trade Co. Ltd, Korea). We analyzed three mitochondrial DNA markers: Cytochrome c oxidase subunit I (COI), NADH dehydrogenase subunit four (ND4), and cytochrome *b* (Cyt*b*). PCR was then performed using an MJ Mini Thermal Cycler PTC-1148 (Bio-Rad) in mixtures consisting 1 μL of genomic DNA (ca. 100 ∼ 200 ng), 2 μL of 10× PCR buffer, 1.6 μL of 2.5 mM dNTPs, 1 μL of each primer (10 pmol), 0.1 μL of TaKaRa EX-*Taq* polymerase (TaKaRa Bio Inc., Kyoto, Japan), and distilled water to bring the final volume to 20 μL. PCR products were amplified using universal and species-specific designed primers (Table S1). The PCR profile for the mtDNA COI consisted of initial denaturation at 94 °C for 4 min, followed by 34 cycles of denaturation at 94 °C for 30 s, annealing at 52 °C for 30 s, extension at 72 °C for 30 s, and a final extension at 72 °C for 8 min; for the mtDNA ND4 gene consisted of initial denaturation at 94 °C for 3 min, followed by 34 cycles of denaturation at 94 °C for 30 s, annealing at 60 °C for 1 min, extension at 72 °C for 5 min, and a final extension at 72 °C for 10 min; and for the mtDNA Cyt*b* consisted of initial denaturation at 94 °C for 5 min, followed by 35 cycles of denaturation at 94 °C for 30 s, annealing at 58–60 °C for 1 min, extension at 72 °C for 1 min, and a final extension at 72 °C for 7 min. The PCR products were purified using a Davinch™ PCR Purification Kit (Davinch-K Co. Ltd, Seoul, Korea). The purified products of mitochondrial DNA were sequenced based on the Sanger sequencing method with an Applied Biosystems ABI 3730XL analyzer (Applied Biosystems, Foster City, CA) using an ABI PRISM BigDye^TM^ Terminator Cycle Sequencing Ready Reaction Kit v3.1 (Applied Biosystems). The sequences were aligned using ClustalW (Thompson et al. 1994) in BioEdit version 7 (Hall 1999).

Genetic diversities were evaluated using Arlequin 3.5.1.2 (Excoffier et al. 2005). To quantify the genetic differences among the six locations, pairwise *Φ*_ST_ values were calculated with 10,000 permutations. Phylogenetic trees were constructed: Neighbor-joining (NJ) and Bayesian inference (BI). The genetic distances were calculated using the Kimura 2-parameter (K2P) model (Kimura 1980) with Mega 6 (Tamura et al. 2013), which showed that the intraspecific genetic distance, and confidence was assessed with 1000 bootstrap replications. The best-fit model of sequence evolution in BI was selected using MrModeltest 2.3 (Nylander 2004). BI was constructed using BEAST 2.5.1 (Drummond and Bouckaert 2015), and importing data and specifying the evolutionary models were done using BEAUti (Drummond et al. 2012). The Markov Chain Monte Carlo (MCMC) analysis of region was run for 10 million generations. The consensus tree along with posterior probabilities was visualized using FigTree Ver. 1.4.3. (Rambaut 2016). The numbers on the nodes indicated posterior probability in the BI. We also performed an analysis of molecular variance, hierarchical AMOVA (analysis of molecular variation) using Arlequin 3.5.1.2. (Excoffier et al. 1992). It was performed to test the best hierarchical groupings based on three alternative hypotheses in geographic structure. The significance of the observed variances for each hierarchical comparison was tested by 10,000 permutations. The haplotype sequences of each region have been deposited with GenBank (MN921151-MN921161, COI; MN965669-MN965691, ND4; MN965641-MN965668, Cyt*b*).

## Results and discussion

### Sequence variation and genetic diversity

All populations displayed various numbers of mitochondrial haplotypes, with a total 11 haplotypes detected among 124 individuals for COI, 23 haplotypes detected among 180 individuals for ND4, and 28 haplotypes detected among 152 individuals for Cyt*b* (Table S2). Analyses of 1852 bp of the combined mtDNA (COI, ND4, and Cyt*b*) identified 43 haplotypes detected among 112 individuals from six locations (Table S2). In the combined dataset, HP1 and HP3 were the most abundant haplotypes, appearing 19 and 11 times, respectively. Ten haplotypes (HP34 to HP43) were shared by only Shikoku Island. Compared molecular diversities, the Korean *E. burgeri* generally showed higher haplotype diversity (0.8696–1.0000) and nucleotide diversity (0.001–0.003) than the Japanese one (0.6209–0.9381, 0.000–0.003, respectively) based on combined mtDNA (Table 1). In case of the brown hagfish, *Eptatretus atami*, they were genetically different in the eastern and the western coast of Japan and their genetic diversity indicated that genetic diversity of *E. atami* in the eastern coast of Japan was higher than those of *E. atami* in the western coast of Japan (Kase et al. 2018). When compared between lampreys and hagfishes which are cyclostomata, Mateus et al. (2013) reported that *Lampetra fluviatilis* (lamprey) showed high haplotype diversity and low nucleotide diversity, and revealed that the existence of four highly divergent allopatric groups within the Iberian Peninsula. The genetic diversity of *E. burgeri* in Kyoto was the lowest, but those of other locations showed higher molecular diversities. Density stratification in the water column occurred in the western coast of Japan during the LGM, and have resulted in serious anaerobic water columns at the sea bottom (Itaki et al. 2004). Thus, *E. burgeri* in Kyoto might have experienced extinction and recolonization in anoxic bottom condition during glacial and interglacial cycle. Some deep-sea species might have experienced a rapid decrease in anaerobic or hypoxia environment after the western coast of Japan was separated from Pacific Ocean during the LGM, which might have influenced their low genetic diversity (Adachi et al. 2009; Sakuma et al. 2014; Habib et al. 2015).

**Table 1.** Summary of genetic variability in the four lineages and two groups of *Eptatretus burgeri* based on the combined mtDNA (COI + ND4 + Cyt*b*).

	n	N	*S*	*h*	Π
Lineage					
I	45	20	19	0.8949 ± 0.0294	0.001451 ± 0.000856
II	50	13	15	0.8057 ± 0.0419	0.001190 ± 0.000725
III	4	7	7	1.0000 ± 0.1768	0.001843 ± 0.001392
IV	13	6	7	0.8462 ± 0.0854	0.000949 ± 0.000644
Group					
A	95	33	36	0. 9234 ± 0.0145	0.003645 ± 0.001901
B	17	10	12	0.8382 ± 0.0867	0.002033 ± 0.001206

n: number of specimens (combined mtDNA COI + ND4 + Cytb); N: number of haplotypes; *S*: polymorphic sites; *h*: haplotype diversity; π: nucleotide diversity.

### Phylogenetic analysis and population structure

**Figure 1.**
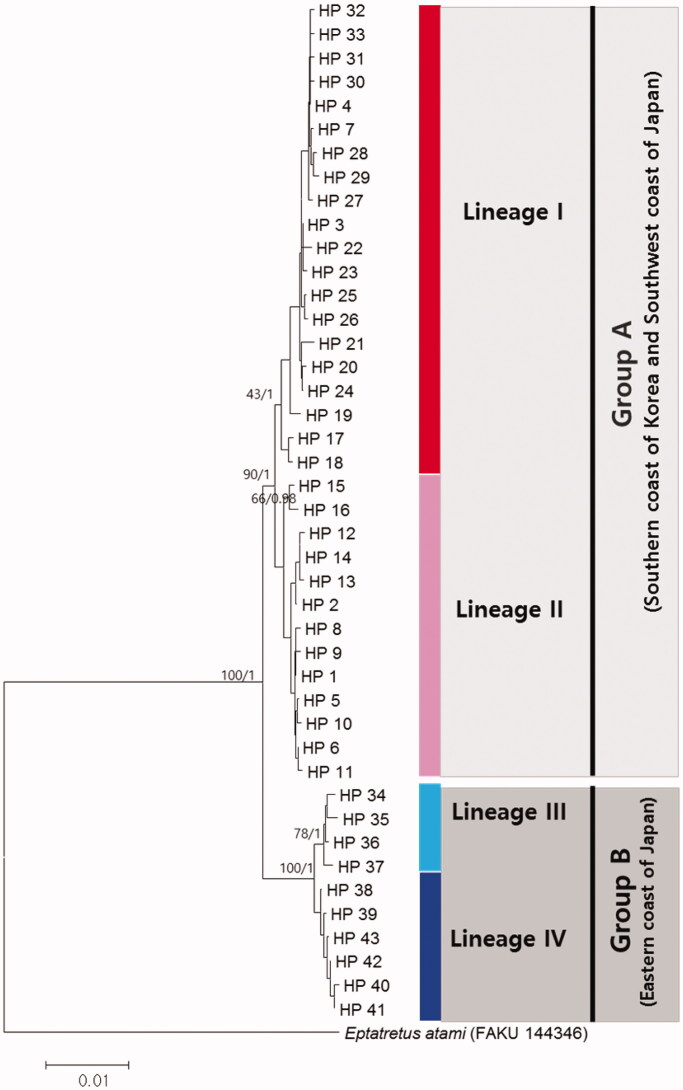
The phylogenetic tree based on the neighbor-joining and Bayesian Inferences for the 43 combined mtDNA region (COI + ND4 + Cyt*b*) haplotypes of *Eptatretus burgeri. Eptatretus atami* was chosen as an outgroup. The numbers on the node is bootstrap value (%) by 1000 replicates in Neighbor-joining methods and posterior probability by Bayesian inferences.

*Eptatretus burgeri* were geographically structured in two distinct groups (Groups A and B) in the northwest Pacific (Figure 1). The genetic distances between Group A and B were 1.1–1.6%, while the within-groups distances were 0.3–0.4% based on combined DNA (COI + ND4 + Cyt*b*). Group A included *E. burgeri* collected from southern coast of Korea and western coast of Japan, whereas Group B included *E. burgeri* collected from the eastern coast of Japan. Group A is geographically divided into two lineages; lineage I which is mainly detected in west-southern coast of Korea, and lineage II which is mainly detected in east-southern coast of Korea and western coast of Japan. Group B also consists of two lineages, lineage III, and lineage IV (Figure 1).

Most *Φ*_ST_ population pairwise comparisons were significant (*p* < .01) except for the *Φ*_ST_ value between Tongyeong and Tsushima Island, and between Jeju Island and Wando Island (Table S3). The pairwise *Φ*_ST_ values between Shikoku Island and the other five locations were especially high than the values among the others (Table S3). The highest *Φ*_CT_ value, which indicates the highest proportion of genetic variance able to be attributed to groups given the present data, was observed as 0.684 (*p* < .000) when the locations were grouped into following two groups: (TY&JJ&WD&TS&KT) and SK (Table 2). Shikoku Island (SK) showed significant genetic differentiation and no shared haplotypes between other locations, indicating no gene flow between two groups. Generally, hagfishes tend to live and breed locally (Walvig 1963) as well as a unique reproductive cycle (e.g. no pelagic larvae) and a lack of migration (Patzner 1978). Therefore, their biological trait and no gene flow might have facilitated allopatric speciation of group A and B of hagfish in the quite different marine environment. In case of benthic fish, the restricted gene flow between the western and eastern coast of Japan of *Okamejei kenojei* have been derived from constrains of low dispersal because of large benthic egg capsules and the absence of a pelagic larval stage (Misawa et al. 2018). Wando Island and Jeju Island (west) are effectively separated from Tongyeong and Tsushima Island (east) by the Seomjin River. Wando Island was only included in lineage I for the COI and the combined mtDNA, and Kyoto was only included in lineage II for all locations except for the Cyt*b* (Figure S2). The different proportion of lineage I to lineage II between the west and the east might have been caused by this low-salinity boundary. In the past, the incised-channel system of the paleo-Seomjin River has significantly affected the sedimentary processes of the southern sea of Korea (Kim et al. 2019). Actually, the two lineages of *Ammodytes japonicus* may be influenced from physical barriers of halocline caused by the Nakdong River in Korea (Kim et al. 2015; Kim et al. 2017). Therefore, the presence of a strong physical barrier and low salinity water column may act as a biogeographical boundary between West and East.

**Table 2.** AMOVA for *E. burgeri* based on the combined mtDNA (COI + ND4 + Cyt*b*) haplotypes from six local populations allocated into two, three or four groups.

Source of variation	*d.f*	Sum of squares.	Variance components	Percentage of variation	Fixation indices
Analysis 1 = ((TY&JJ&WD&TS&KT) & SK)					
Among groups	1	279.868	8.74163	68.44	*Φ*_CT_ : 0.68443 (*p*=.00000)
Among populations within groups	4	111.419	1.40274	10.98	*Φ*_SC_ : 0.34803 (*p*=.00000)
Within populations	106	278.543	2.62777	20.57	*Φ*_ST_ : 0.79426 (*p*=.00000)
Analysis 2 = ((TY&JJ&WD&TS) & KT & SK)					
Among groups	2	331.014	5.39121	59.82	*Φ*_CT_ : 0.59822 (*p*=.00000)
Among populations within groups	3	60.272	0.9931	11.02	*Φ*_SC_ : 0.27427 (*p*=.00000)
Within populations	106	278.543	2.62777	29.16	*Φ*_ST_ : 0.70842 (*p*=.00000)
Analysis 3 = ((TY&TS) & (JJ&WD) & KT & SK)					
Among groups	3	383.36	4.76296	63.67	*Φ*_CT_ : 0.6367 (*p*=.00000)
Among populations within groups	2	7.927	0.08996	1.2	*Φ*_SC_ : 0.0331 (*p*=.07722)
Within populations	106	278.543	2.62777	35.13	*Φ*_ST_ : 0.64873 (*p*=.00000)

TY: Tongyeong, Korea; JJ: JeJu Island, Korea; WD: Wando Island, Korea; KT: Kyoto, Japan; TS: Tsushima Island, Japan; SK: Shikoku Island, Japan.

The distribution pattern of hagfish could reflect the habitat preferences (TNC 2019). Because hagfishes tend to prefer a habitat of mud or muddy sand bottom (Fernholm 1974), environmental change including topographic change might affect the distribution and genetic structure of hagfishes. The temperature in the southern coast of Korea have lower than the western coast of Japan, and the bottom substrate of the southern coast of Korea is made of a higher proportion of sand and muddy than those of the western coast of Japan (Yoo et al. 2014). Therefore, high adaptation to habitat might have resulted in higher genetic diversities in the Korean *E. burgeri* than the Japanese one. In demersal and deep-sea fish similar to the habitat trait of hagfish, another reason to affect gene flow may consider the biogeographical barrier. *Lycodes matsubarai* indicated the high genetic population structure because they have been restricted post-glacial migration between western coast of Japan and Sea of Okhotsk by the shallow strait (13–55 m depth) (Sakuma et al. 2014).

Some studies have revealed that genetic groups or lineages with high divergence could be considered a complex of incipient or cryptic resident species, which allows the definition of evolutionary significant unit (ESU) and management unit (MU) (Ryder 1986; Mateus et al. 2013; Guo et al. 2014). Considering the apparent genetic divergence and different genetic diversity between the two groups, we proposed that the *E. burgeri* in the southern coast of Korea and the western coast of Japan should be recognized as distinct ESU differing from the *E. burgeri* in the eastern coast of Japan. In addition, the *E. burgeri* in southern coast of Korea should be recognized as distinct MU differing from the *E. burgeri* in the western coast of Japan.

## Supplementary Material

Supplemental Material

## Data Availability

The data that support the findings of this study are openly available in [NCBI] at [https://www.ncbi.nlm.nih.gov/], reference number [MN921151-MN921161; MN965669-MN965691; MN965641-MN965668]. The other data generated or analyzed during this study are included in this published article and its supplementary information files.
