# Supplementary material for: Cryptic diversity in the inshore hagfish, Eptatretus burgeri (Myxinidae, Pisces) from the northwest Pacific
Source: Mitochondrial DNA B Resour. 2020 Sep 22;5(3):3410–4. doi: 10.1080/23802359.2020.1823256 (PMC7782252; doi:10.1080/23802359.2020.1823256)
Supplement: Supplemental Material [file TMDN_A_1823256_SM5867.docx]

**Appendices**

**Table S1.** Primers used in this study

| Region | Primer Name | Primer Sequence (5’-> 3’) | Reference |
| --- | --- | --- | --- |
| Cyt*b*^1)^ | YSCYTBHag-F | CTT TCG ACT CCA CAT GAC CAA A | Present study |
|  | YSCYTBHag-R | AGG GGT ATA GCT CCA AGT CAG G | Present study |
| ND4^2)^ | ND4-Hag-F | GAT TAG GTT GTT TTT CTG CCC TAC | Present study |
|  | ND4-Hag-R | GTG CTG ARA CAG GAG TTG GGC CYT CTA T | Present study |
| COI^3)^ | FishF1 | TCA ACC AAC CAC AAA GAC ATT GGC AC | Ward et al. (2005) |
|  | FishR1 | TAG ACT TCT GGG TGG CCA AAG AAT CA | Ward et al. (2005) |

Mitochondrial DNA maker abbreviations: **^1)^** cytochrome *b* (Cyt*b*), **^2)^** NADH dehydrogenase subunits 4 (ND4), and **^3)^** cytochrome C oxidase subunits I (COI)

**Table S2.** Genetic diversity based on various mitochondrial DNA markers for six *Eptatretus burgeri* populations from each location in northwestern Pacific.

| Genetic maker | Locality | n | N | *h* | π |
| --- | --- | --- | --- | --- | --- |
| MtDNA COI | Tongyeong, Korea | 30 | 3 | 0.6000±0.0657 | 0.002492±0.001783 |
|  | Jeju Island, Korea | 24 | 7 | 0.5036±0.1226 | 0.001773±0.001411 |
|  | Wando Island, Korea | 8 | 2 | 0.5714±0.0945 | 0.001093±0.001118 |
|  | Kyoto, Japan | 20 | 3 | 0.4158±0.1157 | 0.000835±0.000872 |
|  | Tsushima Island, Japan | 25 | 4 | 0.6167±0.0639 | 0.002282±0.001684 |
|  | Shikoku Island, Japan | 17 | 2 | 0.3824±0.1132 | 0.000731±0.000812 |
|  | All localities | 124 | 11 | 0.7637±0.0247 | 0.003974±0.002478 |
| MtDNA ND4 | Tongyeong, Korea | 38 | 12 | 0.8649±0.0303 | 0.003108±0.001852 |
|  | Jeju Island, Korea | 33 | 6 | 0.4205±0.1027 | 0.001688±0.001145 |
|  | Wando Island, Korea | 30 | 6 | 0.3632±0.1109 | 0.000704±0.000621 |
|  | Kyoto, Japan | 31 | 1 | 0.0000±0.0000 | 0.000000±0.000000 |
|  | Tsushima Island, Japan | 30 | 8 | 0.8368±0.0367 | 0.003363±0.001992 |
|  | Shikoku Island, Japan | 18 | 6 | 0.7582±0.0701 | 0.001516±0.001085 |
|  | All localities | 180 | 23 | 0.7809±0.0220 | 0.005522±0.003077 |
| MtDNA Cyt*b* | Tongyeong, Korea | 36 | 9 | 0.8016±0.0413 | 0.005336±0.003123 |
|  | Jeju Island, Korea | 33 | 10 | 0.7803±0.0482 | 0.004242± 0.002588 |
|  | Wando Island, Korea | 13 | 5 | 0.7436±0.0866 | 0.003241± 0.002194 |
|  | Kyoto, Japan | 27 | 4 | 0.2137±0.1033 | 0.001284± 0.001069 |
|  | Tsushima Island, Japan | 25 | 6 | 0.7667±0.0667 | 0.004765±0.002878 |
|  | Shikoku Island, Japan | 18 | 8 | 0.6993±0.1171 | 0.002987± 0.002010 |
|  | All localities | 152 | 28 | 0.8367±0.0170 | 0.008052±0.004360 |
| Combined MtDNA | Tongyeong, Korea | 29 | 16 | 0.9138±0.0398 | 0.003642±0.001948 |
| (COI+ND4+Cyt*b*) | Jeju Island, Korea | 24 | 13 | 0.8696±0.0515 | 0.002113±0.001205 |
|  | Wando Island, Korea | 3 | 3 | 1.0000±0.2722 | 0.001603±0.001393 |
|  | Kyoto, Japan | 18 | 6 | 0.6209±0.1211 | 0.000497±0.000418 |
|  | Tsushima Island, Japan | 21 | 12 | 0.9381±0.0303 | 0.003411±0.001861 |
|  | Shikoku Island, Japan | 17 | 9 | 0.9118±0.0561 | 0.002022±0.001182 |
|  | All localities | 112 | 43 | 0.9414±0.0110 | 0.006338±0.003202 |

n, number of specimens; N, number of haplotypes; *h*, haplotype diversity; π, nucleotide diversity.

**Table S3.** Pairwise *Φ*_ST_ values for the various mitochondrial DNA regions among six populations of *Eptatretus burgeri*.

| **Genetic region** | Locality | TY | JJ | WD | KT | TS |
| --- | --- | --- | --- | --- | --- | --- |
| MtDNA COI | JJ | 0.24293* |  |  |  |  |
|  | WD | 0.56281* | 0.51644* |  |  |  |
|  | KT | 0.26097* | 0.68236* | 0.85769* |  |  |
|  | TS | -0.02126 | 0.31956* | 0.63482* | 0.21768* |  |
|  | SK | 0.71208* | 0.83552* | 0.92289* | 0.83439* | 0.72402* |
| MtDNA ND4 | JJ | 0.17287* |  |  |  |  |
|  | WD | 0.34380* | 0.04798 |  |  |  |
|  | KT | 0.48677* | 0.80935* | 0.93485* |  |  |
|  | TS | 0.00743 | 0.27866* | 0.45455* | 0.37224* |  |
|  | SK | 0.75518* | 0.84190* | 0.90119* | 0.94979* | 0.75574* |
| MtDNA Cyt*b* | JJ | 0.18271* |  |  |  |  |
|  | WD | 0.24853* | 0.00214 |  |  |  |
|  | KT | 0.25627* | 0.58228* | 0.73572* |  |  |
|  | TS | 0.02799 | 0.30376* | 0.39332* | 0.10906* |  |
|  | SK | 0.7613* | 0.80645* | 0.83109* | 0.89033* | 0.78585* |
| Combined MtDNA | JJ | 0.25400* |  |  |  |  |
| (COI+ND4+Cyt*b*) | WD | 0.26894** | 0.07647 |  |  |  |
|  | KT | 0.28431* | 0.69513* | 0.81686* |  |  |
|  | TS | 0.01265 | 0.38848* | 0.41037** | 0.15133* |  |
|  | SK | 0.74300* | 0.83582* | 0.85378* | 0.87491* | 0.76431* |

Significant *P* values are indicated * P < 0.01 and ** P < 0.05. TY, Tongyeong, Korea; JJ, JeJu Island, Korea; WD, Wando Island, Korea; KT, Kyoto, Japan; TS, Tsushima Island, Japan; SK, Shikoku Island, Japan.

**.
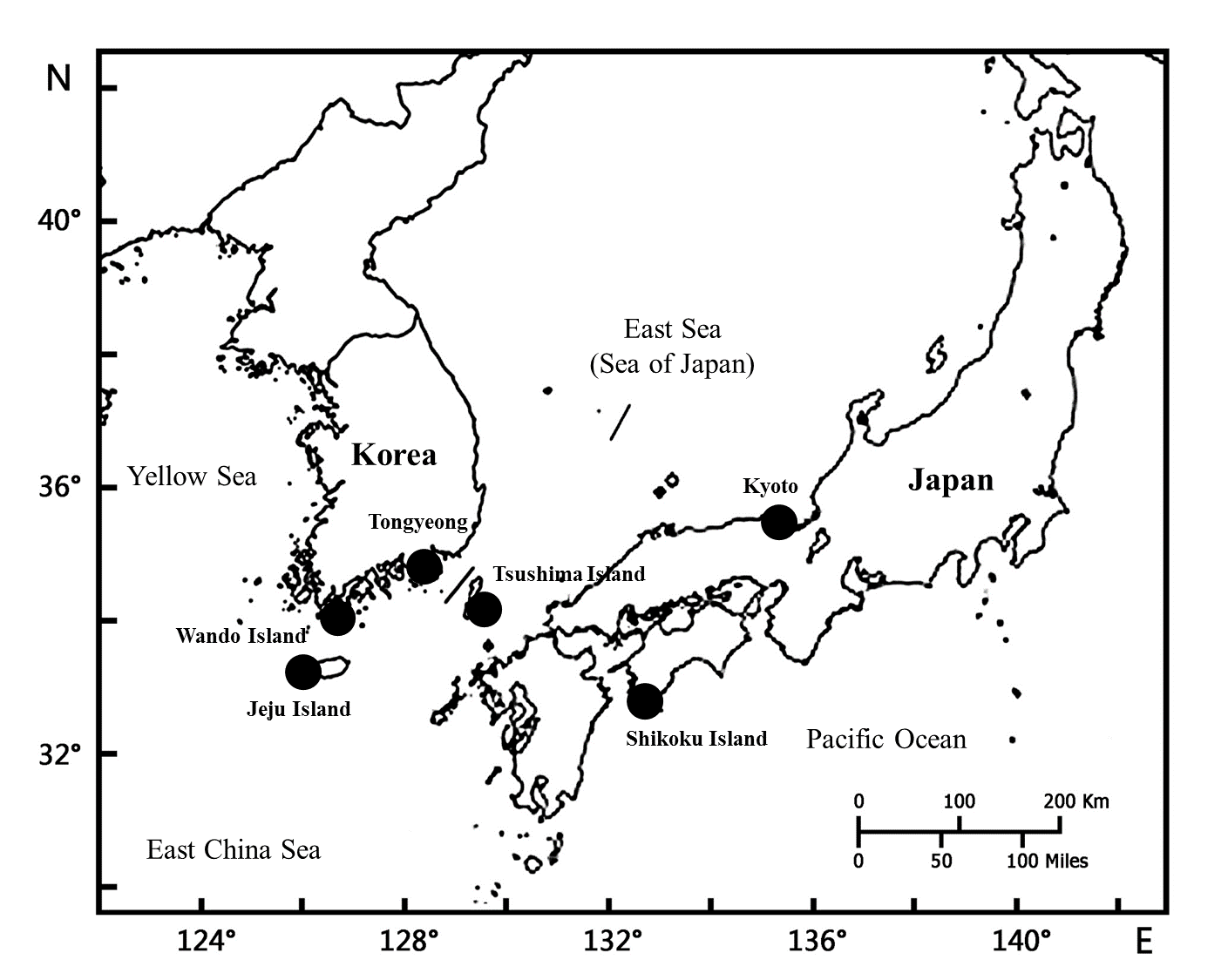
**

**Figure S1.** Map showing the sampling sites of inshore hagfish in the northwestern Pacific Ocean.


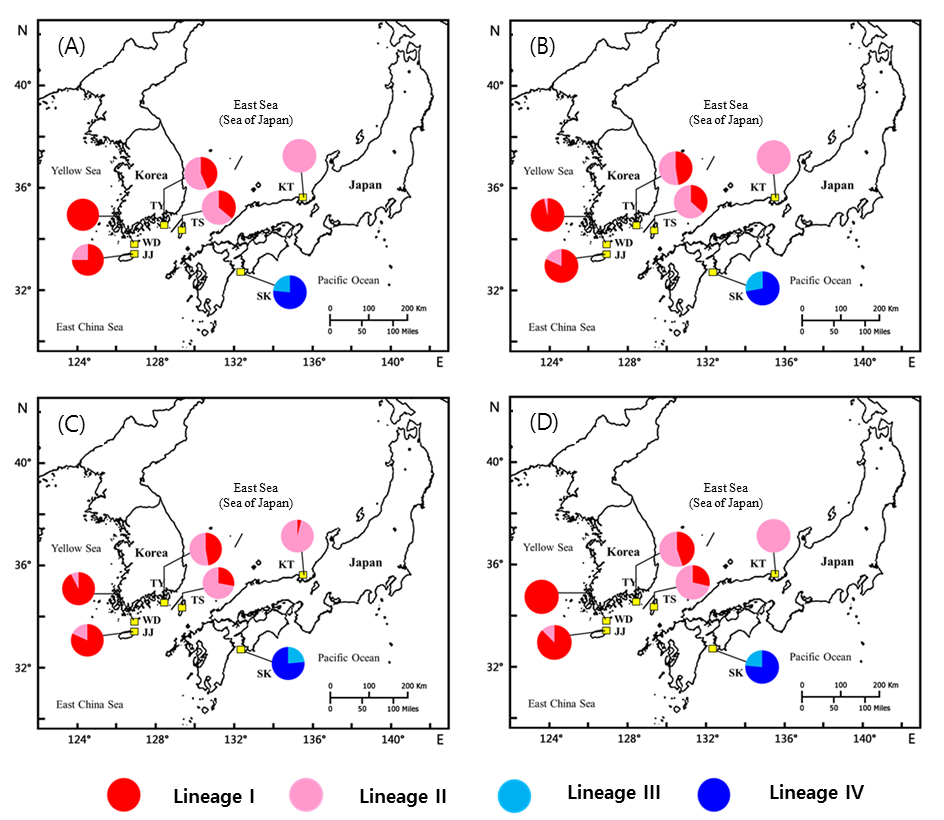


**Figure S2.** Geographical distribution of (A) mtDNA COI, (B) mtDNA ND4, (C) mtDNA Cyt*b*, and (D) the combined mtDNA (COI+ND4+Cyt*b*) haplotypes fixed on four lineages. Abbreviation: TY, Tongyeong; JJ, Jeju Island; WD, Wando Island; KT, Kyoto; TS, Tsushima Island; SK, Shikoku Island.
